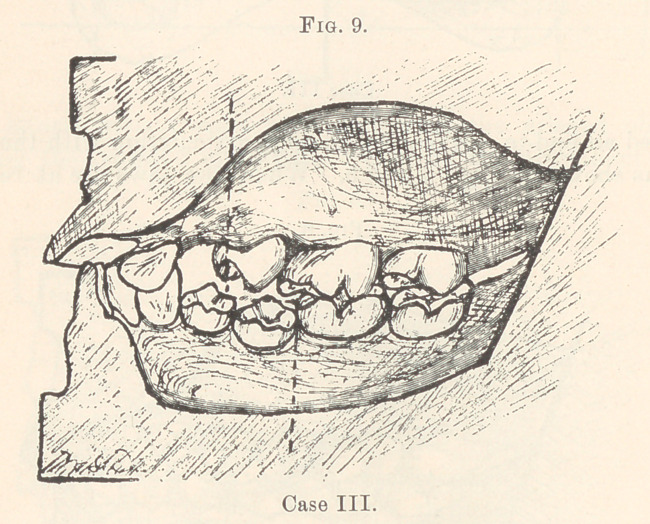# Regulating without Extraction *Versus* Extraction for Regulating; Some Typical Comparative Results

**Published:** 1897-10

**Authors:** Wm. Slocum Davenport

**Affiliations:** Paris, France


### International Dental Journal.

Vol. XVIII. October, 1897. No. 10.

### Original Communications.[1]

### REGULATING WITHOUT EXTRACTION *VERSUS* EXTRACTION FOR REGULATING; SOME TYPICAL COMPARATIVE RESULTS.

BY WM. SLOCUM DAVENPORT, D.D.S., PARIS, FRANCE.

Case I.—Figs. 1 and 2 represent the mouth of an English youth sixteen years of age. By examining the position of the teeth by the aid of the superposed cross-lines one sees that the true median line, in Fig. 1, would pass through the left central incisor, three millimetres to the left, and that the central right lateral, left canine, and left bicuspids stand far within the normal arch, while the right canine stands out of the arch and the right bicuspids are nearly normal.

The first means employed towards correcting the irregularity was to adjust a band to the canine; a "Coffin W-plate" was arranged with linen ligatures doubled and tied at the left side of the split, as shown in Fig. 3. The patient was able to loop the free end of the ligature over the hook at the front of the band on the canine, when by rotating the plate (still out of the mouth) the ligature was twisted until sufficient tension was secured to furnish the degree of force desired, when the plate was pressed into

---

[1] The editor and publishers are not responsible for the views of authors of papers published in this department, nor for any claim to novelty, or otherwise, that may be made by them. No papers will be received for this department that have appeared in any other journal published in the country.

position. The effect of the force thus applied was to turn the canine into line, move the centrals and laterals forward, and move

Figs. 1 and 2.

Case I.

all the front teeth to the left, restoring the relation of the teeth to the median line. By comparing the measurements of Figs. 1 and

2 by *a*, *a* and *B*, *B*, we find that the relative distance between the canines is the same, but that the six front teeth have been carried

Fig. 3.

Case I.

to the left three millimetres, thus correcting the median as well as the general deviation. I saw this patient not more than twice a

Fig. 4.

Case II.

week for two months, during which time the lower arch also was spread to conform with the upper arch.

Case II.—Figs. 4, 5, and 6, represent the mouth of the writer at the age of fourteen. The left upper second bicuspid and the right

Fig. 5.

Case II.

lower lateral incisor were extracted with the alleged object of relieving the crowded arches and "to allow the prominent canine

Fig. 6.

Case II.

to grow down." The space in the upper jaw left by the extracted tooth was closed partly by the dropping backward of the canine and partly by the moving forward of the second bicuspid and molars. The new position taken by the bicuspid and molars

Fig. 7.

Case III.

produced an end-to-end or edge-to-edge articulation with the lower teeth, as shown in Figs. 4 and 5. When the jaws are at rest, the

Fig. 8.

Case III.

teeth on both sides of the mouth strike evenly, but with the lateral motions the cusps (*a*) of the left side are first brought into action, opening the bite and preventing a large proportion of contact on the first plane of articulation, as described by I. B. Davenport in his papers upon the "Dental Arches of Man," *Dental Cosmos*, 1887, p. 417, and "Articulation of the Teeth," etc., INTERNATIONAL DENTAL JOURNAL, 1892, p. 7. The result is that the cusps (*a*) are much worn, and so sensitive at times as to render the use of the left side difficult and painful. Fig. 6 shows the right side of the mouth, the articulation being nearly normal; the teeth show no wear, and consequently are not sensitive, still the lower arch is a little contracted, owing to the loss of the central incisor.

CASE III.—Figs. 7, 8, and 9 represent the mouth of a young girl, fifteen years of age. At the age of thirteen a well-known dentist extracted the first right superior bicuspid "to allow the canine to grow into line," and after finding that the canine did not change its position, he even suggested that it might be best to extract the second superior bicuspid of the same side.

It is easy to see in Fig. 7 that the canines are in their normal position. The second bicuspid and molars on the right side have

FIG. 9.

Case III.

moved forward and stand far within the arch. Fig. 8 shows the ideal "interlocking articulation" as it exists on the left side. Fig. 9 illustrates the ruin of the articulation of the right side of the mouth, caused by the removal of the bicuspid, which was needed to hold the arch in shape, but the absence of which has allowed the contraction of the arch and forward displacement of the back teeth.

This is one case from a collection of many I can show where dentists attempt to regulate the wrong tooth, making the case most difficult if not impossible. Let me say to those who extract "to give room," aid nature, and let "interlocking articulation" be your guide. Extraction for crowded arches should be a thing of the past. More attention should be given the sixth-year molars and their proper relation with each other, and if *that* is secured at an early date, crowded arches will become manageable and fewer deformed faces will be the result.

(To be continued.)

## Figures and Tables

**Figs. 1 and 2. f1:**
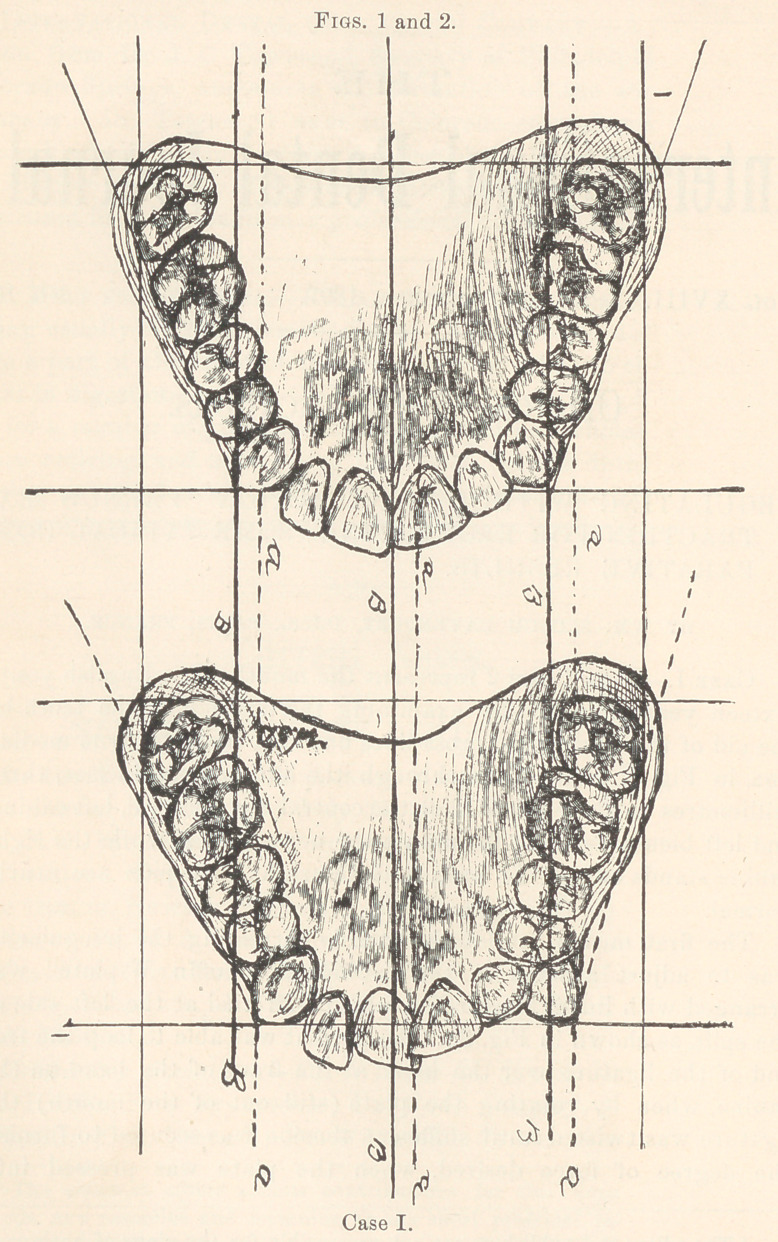


**Fig. 3. f2:**
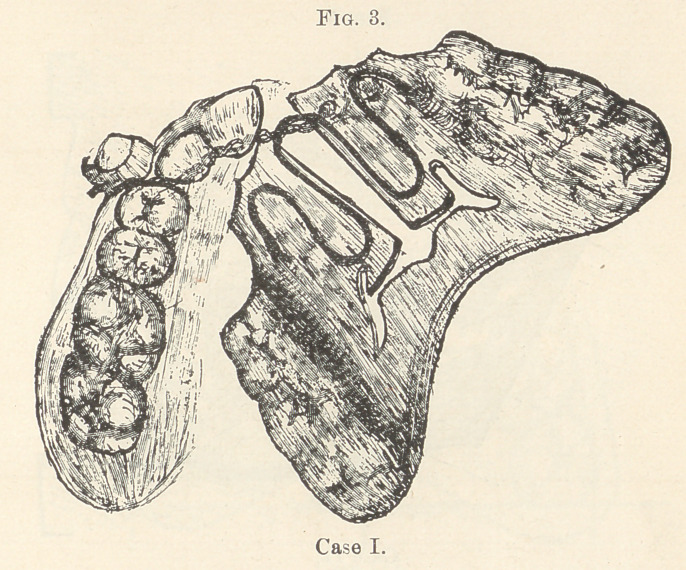


**Fig. 4. f3:**
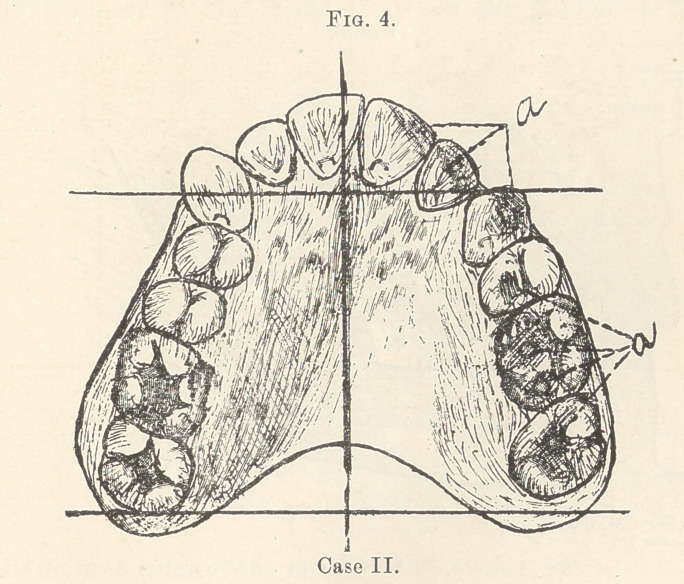


**Fig. 5. f4:**
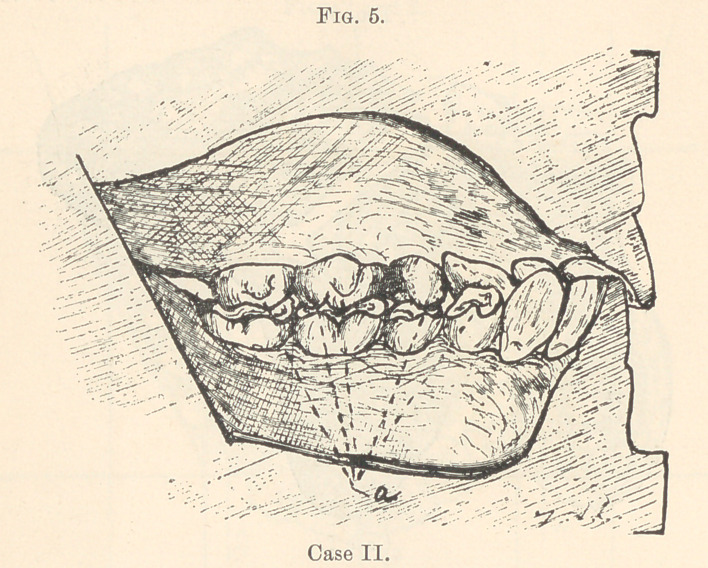


**Fig. 6. f5:**
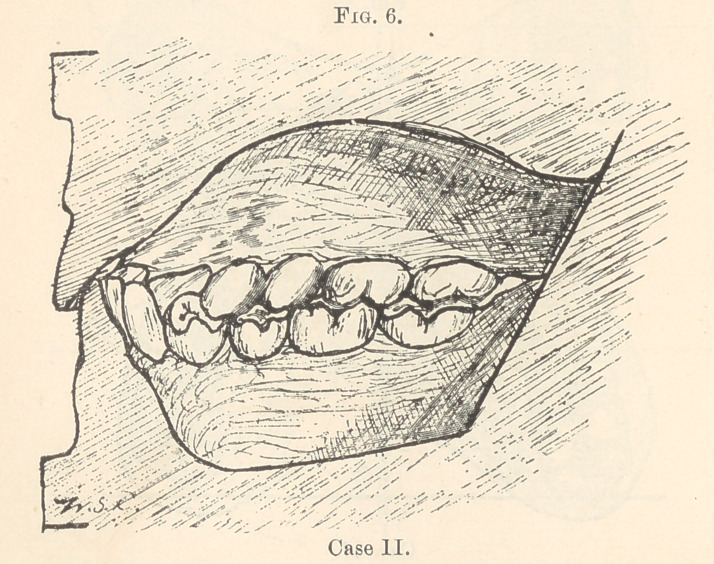


**Fig. 7. f6:**
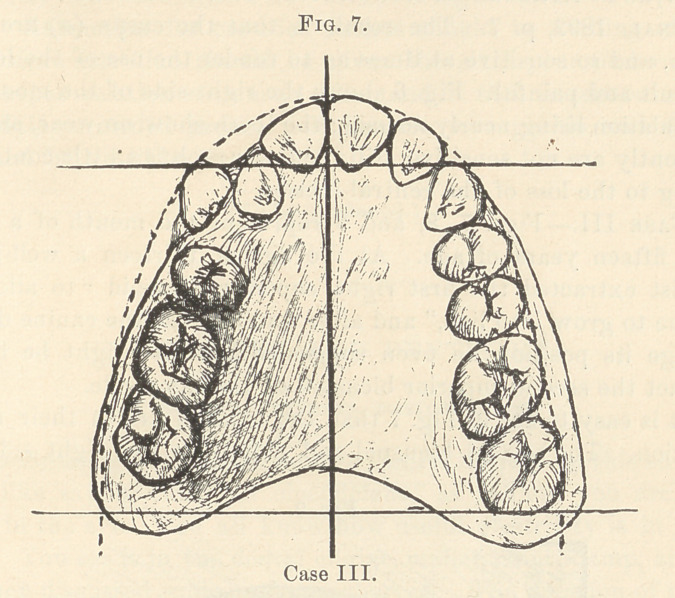


**Fig. 8. f7:**
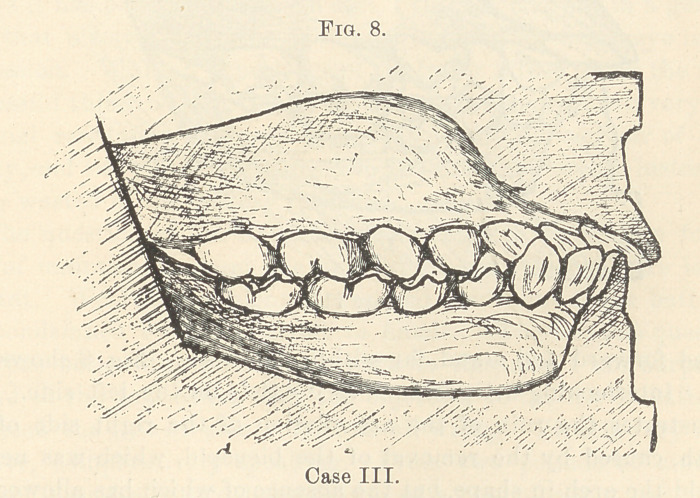


**Fig. 9. f8:**