# Correction

**Published:** 1897-12

**Authors:** 


### CORRECTION.

In the preparation of Dr. Davenport's article in the October number, an error occurred in the preparation for the press in reversing Figs. 1 and 2. They will, however, be readily understood as they stand upon the page.

